# Dose-dependent effects of a recombinant gonadotropin-releasing hormone vaccine on reproductive function and growth performance in male goats

**DOI:** 10.5713/ab.250931

**Published:** 2026-03-11

**Authors:** Fuqiang Pan, Mengdi Han, Wei Qian, Yuke Jia, Hongyan Liao, Weina Li, Ziyi Zheng, Ruonan Yuan, Chunyan Yuan, Fugui Fang, Yunsheng Li, Yinghui Ling, Hongwei Duan, Ya Liu

**Affiliations:** 1 College of Veterinary Medicine, Anhui Agricultural University, Hefei, China; 2 College of Veterinary Medicine, Nanjing Agricultural University, Nanjing, China; 3 Anhui Divinity Biological Products Co., Ltd., Bozhou, China; 4 College of Animal Science and Technology, Anhui Agricultural University, Hefei, China

**Keywords:** Gonadotropin-releasing Hormone Vaccine, Growth Performance, Immunocastration, Male Goats, Reproductive Suppression

## Abstract

**Objective:**

This study aimed to develop a recombinant gonadotropin-releasing hormone (GnRH) vaccine and to investigate the effects of different immunization doses on immunocastration efficacy and growth performance in male goats; thererby providing an animal welfare-friendly alternative to surgical castration (SC).

**Methods:**

Forty male goats were randomly assigned to four groups (n = 10 per group): the saline-injected control (NC), SC, low-dose vaccine (LD), and high-dose vaccine (HD). The vaccine was given in accordance with a regular immunization protocol. Anti-GnRH antibody titers, serum testosterone concentrations, libido scores, histological characteristics of testicular tissues, and growth performance were measured to assess immunocastration efficacy.

**Results:**

Both LD and HD groups exhibited significantly higher anti-GnRH antibody titers than the NC group (p<0.01). Serum testosterone concentrations and libido scores were markedly reduced in the immunized groups (p<0.01), indicating effective suppression of reproductive function. Histological and quantitative analyses demonstrated pronounced testicular atrophy in immunocastrated goats, characterized by a significant reduction in seminiferous tubule diameter and spermatogenic cell number, as well as the absence of spermatozoa within the lumen. Although only limited time points showed significant differences between the two immunization doses, the overall immunocastration efficacy of the low- and high-dose regimens was largely comparable, with the low-dose group exhibiting slightly stronger suppression in certain parameters or time points. Additionally, immunocastrated goats had significantly higher body weight and weight gain compared to surgically castrated goats (p<0.01), while differences with the NC group were not significant (p>0.05).

**Conclusion:**

The recombinant GnRH vaccine effectively suppresses reproductive function in male goats. Compared with SC, immunocastration was associated with improved growth performance, while no significant differences were observed relative to intact animals. These findings support immunocastration as a non-invasive and animal-friendly alternative to SC in livestock production.

## INTRODUCTION

Castration of male animals is a widely adopted management practice in livestock production, primarily aimed at reducing aggressive and sexual behaviors such as mounting and fighting, thereby improving group housing safety and management efficiency [1]. More importantly, castration can significantly improve meat quality attributes, such as tenderness, uniform fat deposition, and flavor enhancement, thus playing a critical role in improving fattening efficiency and the final product value [2]. Traditional surgical castration (SC), while directly effective, involves procedures such as anesthesia, disinfection, and postoperative care, all of which are associated with considerable stress responses and risks of infection, particularly under intensive farming systems where labor and veterinary resources are limited [3]. Although chemical castration can partially address these issues, it often presents drawbacks such as toxic side effects and limited duration of action, including irritative responses or chronic local inflammation in testicular tissues [4].

Immunocastration, as a non-invasive alternative technology, has attracted increasing attention in the context of animal welfare in recent years. Its principle is to induce animals to produce antibodies against endogenous reproductive hormones, such as gonadotropin-releasing hormone (GnRH), follicle-stimulating hormone (FSH), and luteinizing hormone (LH) or their receptors, through vaccination, thereby disrupting the hormonal feedback mechanism of the hypothalamic–pituitary–gonadal (HPG) axis, ultimately inhibiting gonadal development and sex hormone secretion [5]. This technology is characterized by its simplicity, low stress, and minimal interference with animal growth and development. It is particularly suitable for reproductive control, meat quality improvement, and wild animal population management, and is widely recognized as a promising strategy that balances animal welfare and production efficiency [2].

Currently, GnRH-based vaccines have been commercialized as the primary products for immunocastration, including Improvac for pigs, GonaCon for deer, and Bopriva for cattle and sheep, each achieving favorable reproductive suppression outcomes in their respective target species. For instance, Improvac has been shown to significantly reduce serum testosterone levels and sexual behavior in boars, thereby improving meat flavor and quality [6]. Bopriva effectively reduces sperm density and inhibits testicular development in sheep [7]. However, when applied to other species, these vaccines exhibit notable limitations, including strong species specificity, incomplete reproductive suppression, and unstable immune responses. For example, antibody titers induced by GonaCon decline two to three times faster in goats compared to target species [8].

Currently, there are no vaccines in the market that effectively castrate male goats. Recently, we developed a GnRH octamer vaccine, which effectively inhibits the testicular development of male goats [9]. Accumulating evidence suggests that antigen dosage plays an important role in determining both the magnitude and duration of the immune response. Insufficient doses may fail to elicit an adequate immune response, while excessively high doses carry the risk of inducing immune tolerance [10]. In this study, we systematically evaluated the effects of the GnRH octamer vaccine on testicular development, libido, and growth performance in goats under high-dose (HD) and low-dose (LD) administration regimes, providing essential data to support its future clinical application.

## MATERIALS AND METHODS

### Animals

A total of 40 healthy male Wanlin white goats, aged 3 months and with similar body weights (19.19±0.48 kg), were provided by Tianyuan Animal Husbandry. All animals were housed in a standardized barn under identical management conditions, with free access to water and exposure to natural ventilation and lighting.

### Vaccine construction and preparation

The recombinant GnRH vaccine was constructed using the pMAL-c2X plasmid as an expression vector. The specific GnRH8 antigen sequence [9] was inserted in-frame into the multiple cloning site of pMAL-c2X to generate a fusion protein. The recombinant plasmids were transformed into *Escherichia coli* strain BL21 (DE3), and protein expression was induced with 0.1 mM IPTG. The expressed proteins were purified following the manufacturer’s instructions using a PurKine Maltose Binding Protein Dextrin Resin (BMR2020; Abbkine), and subsequently dialyzed against phosphate-buffered saline (PBS) to remove impurities and endotoxins.

The purified protein was dissolved in physiological saline and mixed with a white oil adjuvant (MONTANIDE ISA 201 VG, U51021; SEPPIC) at a 1:1 (v/v) ratio, followed by thorough emulsification. The emulsified vaccine was administered to goats by muscle injection within 24 h after preparation.

### Experimental design

A total of 40 healthy 3-month-old male goats were randomly assigned to four groups (n = 10 per group): the saline-injected control (NC) group received 1 mL physiological saline intramuscularly; the SC group underwent bilateral orchiectomy via a scrotal incision; the LD and HD immunization groups were injected with 1 mL recombinant GnRH vaccine (3 mg/mL and 6 mg/mL, respectively) into the lateral thigh muscle, followed by booster vaccinations with identical dosages 30 days later.

The vaccine doses were selected based on previous immunocastration studies using GnRH-based vaccines, in which antigen doses within a comparable range were sufficient to induce robust and sustained anti-GnRH antibody responses in livestock species [9]. In our previous study, effective immunocastration in goats was achieved using 1 mg of a His-tagged GnRH8 multimer antigen. In the present study, GnRH8 was expressed as a fusion protein with maltose-binding protein (MBP), resulting in a substantially higher molecular weight compared with the His-tagged construct. Therefore, the LD (3 mg) formulation was selected to provide an antigen amount approximately equivalent to that used in our prior work, when adjusted for fusion tag size. The HD (6 mg) formulation was set at a two-fold higher level to evaluate potential dose-dependent effects on immunogenicity and physiological outcomes.

The experimental period extended from 3 to 8 months of age. During this period, serum testosterone concentrations were measured monthly using chemiluminescence assays (sensitivity: 0.07 ng/mL), anti-GnRH antibody levels were determined by indirect enzyme-linked immunosorbent assay (ELISA) (expressed as OD450 values), and body weight changes were recorded monthly. Libido was evaluated concurrently using a 5-point scoring system.

At the end of the experiment (8 months of age), all animals were humanely slaughtered. Testes were collected for morphological measurements, including longitudinal diameter, transverse diameter, and circumference using a vernier caliper, and testicular weight was measured using an electronic balance. Prior to measurement, all testes were carefully cleared of residual surface tissues and blood, rinsed with PBS, and gently blotted to remove excess moisture to ensure measurement accuracy. For histological analysis, testicular tissues were fixed in 4% paraformaldehyde, embedded in paraffin, sectioned, and stained with hematoxylin and eosin (H&E) to observe structural changes in the seminiferous tubules.

### Antibody detection

The ELISA coating antigen was based on a truncated GnRH construct (GnRH4), which represents an internal fragment of the GnRH8 sequence and retains the repeated GnRH core epitopes. GnRH4 was expressed as a GST fusion protein to facilitate soluble expression and purification, as the full-length GnRH8 construct exhibited predominantly insoluble expression when fused to GST. Importantly, GnRH4 shares identical GnRH repeat motifs with the GnRH8 immunogen, ensuring epitope overlap and allowing specific detection of anti-GnRH antibodies elicited by vaccination. Moreover, the use of different fusion tags for immunization (MBP) and antibody detection (GST) was intended to minimize potential interference from anti-tag antibodies and to improve the specificity and interpretability of the ELISA results.

The recombinant plasmid pGEX-6P-1-GnRH4 (GnRH4 sequence intercepted in mid-GnRH8 [9]) was expressed and purified to obtain the target protein. After determining the protein concentration, the protein was diluted to 2 μg/mL using PBS. Then, 100 μL of the diluted protein solution was added to each well of a 96-well microplate and incubated overnight at 4°C for coating. The next day, the coating solution was discarded, and each well was washed three times with 250 μL of PBST (PBS containing 0.05% Tween-20), followed by tapping dry on absorbent paper.

Subsequently, 200 μL of blocking solution was added to each well and incubated at 37°C for 1 hour. After incubation, the blocking solution was discarded. Serum samples were diluted 1:700 and added to the wells (100 μL per well) in triplicate. The plate was incubated at 37°C for 1 hour, then washed three times with PBST and dried. A rabbit anti-goat IgG (HRP-conjugated) secondary antibody (A21030; Abbkine) was diluted 1:50,000 with blocking solution, mixed thoroughly, and 100 μL was added to each well. After a further 1-hour incubation at 37°C, the plate was washed three times with PBST and dried.

For color development, 100 μL of TMB substrate solution was added to each well and incubated at 37°C in the dark for 15 minutes. Then, 50 μL of stop solution was added to each well. Once the solution color changed completely from blue to yellow, bubbles were carefully removed, and the optical density at 450 nm (OD450) was measured within 15 minutes.

To ensure assay reliability, both positive and negative control sera were included on each plate. Positive control serum was obtained from goats that had previously been immunized with a validated GnRH peptide vaccine [9], whereas negative control serum was collected from untreated goats. Serial two-fold dilutions of the positive control serum (from 1:100 to 1:12,800) were used to assess assay sensitivity and linearity. Antibody titers were defined as the reciprocal of the highest serum dilution yielding an OD450 value exceeding the mean of the negative control plus three standard deviations; however, this parameter was used solely for assay validation. For data presentation and statistical analysis, anti-GnRH antibody responses were quantified using OD450 values measured at the fixed serum dilution. Intra-assay variability was assessed by measuring each sample in triplicate wells within the same plate, and inter-assay variability was evaluated by repeating the assay on three independent occasions. The coefficients of variation for both intra- and inter-assay measurements were below 10%, confirming the precision and reproducibility of the assay.

### Testosterone detection

Testosterone (T) levels were quantified using a chemiluminescence immunoassay (CLIA) (MINDRAY). Briefly, samples were initially mixed with testosterone antibodies labeled with alkaline phosphatase (ALP) and magnetic latex reagents. Subsequently, a chemiluminescent substrate, 3-[2-spiroadaman-tane]-4-methoxy-4-[3-phosphoryloxy]-Phenyl-1,2-dioxetane (AMPPD), was introduced to generate chemiluminescent signals that were quantified by counting the emitted photons. The minimum detection limit is 0.1 ng/mL, with a detection range from 0.1 ng/mL to 16.0 ng/mL, and coefficient of variation (CV) values within the range of 5.82% for intra-assay and 4.22% for inter-assay, respectively. The correlation coefficient of the standard curve is 0.9900.

### Libido scoring system

Based on the standards described in previous studies [11], libido evaluation was performed on goats from the SC, NC, LD, and HD groups starting at 5 months of age. Each assessment lasted for 15 minutes under controlled observation conditions.

The libido scoring system was defined as follows:

• Score 0: No reaction observed after contact with an estrous female goat.
• Score 1: Actively approaches and sniffs or licks the vulva of the estrous female, with the upper lip curling (Flehmen response), but quickly loses interest and enters a refractory phase.
• Score 2: Rapid initiation of sexual arousal upon contact with the estrous female; actively approaches and sniffs or licks the vulva, frequently exhibits “lip-smacking” behavior, occasionally shows penile protrusion without attempting to mount.
• Score 3: Displays a strong sexual response toward the estrous female; approaches while making gurgling sounds, sometimes attempts to mount with penile protrusion, but no ejaculation occurs.
• Score 4: Exhibits normal sexual behavior with high libido, successfully mounts, copulates, and ejaculates during natural mating.

### Testicular tissue section preparation

As described previously [12], the testes were collected and fixed in 4% paraformaldehyde, dehydrated in ethanol, embedded in paraffin wax, sectioned, and stained with H&E staining [13]. The tissue structure was observed under microscope (BX51). Images were taken (Nikon Digital Sight DS-SMC camera) and saved.

For quantitative histological analysis, seminiferous tubule diameter was measured using ImageJ software. For each group, 30 seminiferous tubule cross-sections that appeared nearly circular were randomly selected from H&E-stained sections, and the longest diameter of each tubule was recorded to minimize sectioning-related bias.

Spermatogenic cell number was quantified from H&E-stained sections by counting cells with intact nuclei within seminiferous tubule cross-sections. For each group, 30 nearly circular seminiferous tubules were randomly selected. Spermatogenic cells included primary spermatocytes, secondary spermatocytes, and both immature and mature spermatids. The average cell number per tubule was used for statistical analysis.

### Statistical analysis

All statistical analyses were performed using IBM SPSS Statistics version 24 For parameters measured at a single endpoint (e.g., testis weight, histological parameters), one-way ANOVA was applied to compare differences among groups, followed by Tukey’s post hoc test. For dynamic parameters measured repeatedly over time (e.g., anti-GnRH antibody titers, serum testosterone levels, and body weight curves), a two-way repeated measures ANOVA was conducted, with treatment as the between-subject factor and time as the within-subject factor. When a significant treatment × time interaction was detected, simple effects analyses with Bonferroni correction were performed to examine group differences at each time point. Results are expressed as mean±standard error of the mean (SEM). Statistical significance was defined as p<0.05, and p<0.01 was considered highly significant. Graphs were generated using GraphPad Prism ver. 3.0 (GraphPad Software).

## RESULTS

### Changes in antibody levels

**Figure 1:**
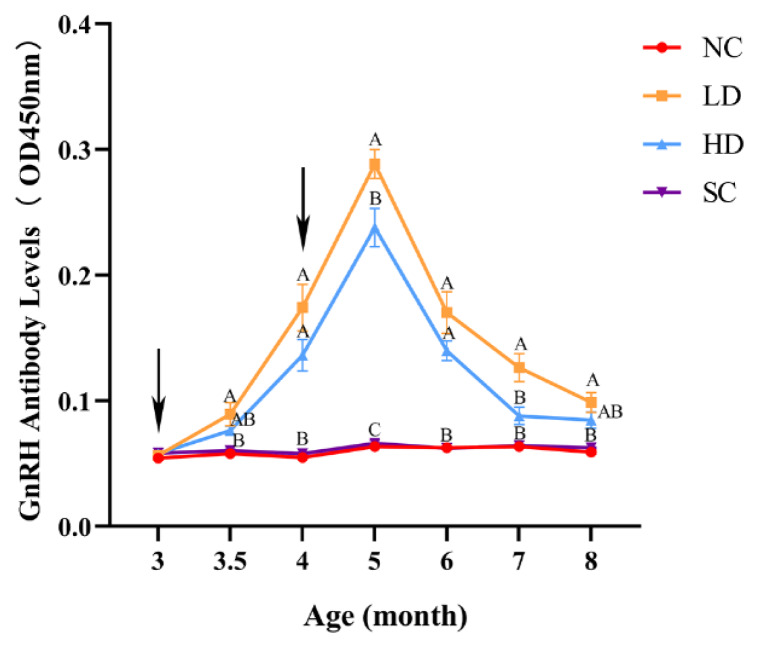
Temporal dynamics of anti-GnRH antibody levels following immunization. Antibody levels were measured by ELISA at a 1:700 dilution, and absorbance was read at 450 nm. Line chart illustrating the changes in serum anti-GnRH antibody titers in the saline-injected control (NC), low-dose (LD), high-dose (HD), and surgical castration (SC) groups. Arrows indicate the timing of immunizations. ^A–C^ Different uppercase letters at the same time point indicate p<0.01; the presence of identical letters indicates p>0.05. GnRH, gonadotropin-releasing hormone; ELISA, enzyme-linked immunosorbent assay.

As shown in Figure 1, immunization with the recombinant GnRH vaccine successfully induced anti-GnRH antibody responses in both the LD and HD groups, whereas antibody levels remained negligible in the NC and SC groups throughout the experimental period. A two-way repeated measures ANOVA revealed a significant treatment × time interaction for anti-GnRH antibody levels expressed as OD450 values (F = 45.79, p<0.01), indicating distinct temporal response patterns among groups.

Detectable antibody levels appeared as early as 15 days after the primary immunization and increased markedly following the booster immunization, reaching peak OD450 values at approximately 5 months of age. Both LD and HD groups exhibited similar temporal patterns of antibody production; however, the LD group showed higher mean OD450 values at certain sampling points, particularly around the peak response period. However, these differences were time-dependent and not consistently observed throughout the entire experimental period.

Together, these results demonstrate that 3 mg of recombinant GnRH protein induced a strong and sustained anti-GnRH antibody response in immunized goats, and increasing the antigen dose does not further enhance the antibody concentration.

### Changes in serum testosterone levels

**Figure 2:**
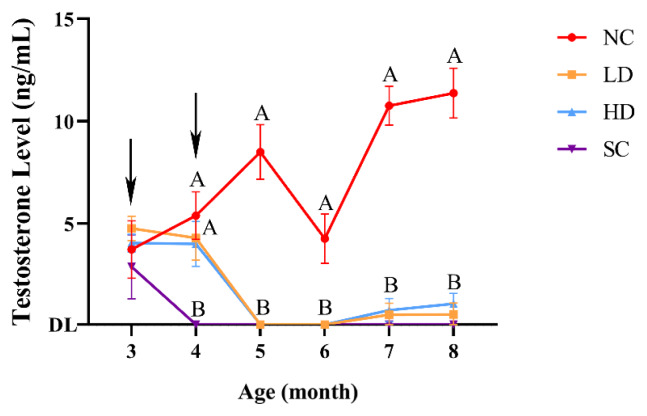
Serum testosterone concentrations following immunization. Serum samples collected at different time points were analyzed using chemiluminescent immunoassay to determine testosterone levels. Saline-injected control (NC), low-dose (LD), high-dose (HD), and surgical castration (SC) groups. Arrows indicate the timing of immunizations. DL represents the detection limit of the assay (0.07 ng/mL). ^A–B^ Different uppercase letters indicate p<0.01; the identical letters indicate p>0.05.

As shown in Figure 2, serum testosterone levels varied significantly among groups over time. A two-way repeated measures ANOVA revealed a significant treatment×time interaction (F = 9.56, p<0.01), indicating that testosterone dynamics differed across treatment groups.

In the SC group, testosterone concentrations dropped below the detection limit (<0.07 ng/mL) from 4 months of age onward. Similarly, in both vaccinated groups (LD and HD), testosterone levels fell below the detection limit after the booster immunization, beginning at 5 months of age, achieving an effect comparable to that of SC. In contrast, the NC group maintained significantly higher testosterone concentrations than the other groups from 5 to 8 months of age (p< 0.01).

A transient decrease in serum testosterone levels was observed in the NC group at 6 months of age. In addition, slight increases in testosterone concentrations were detected in the LD and HD groups at 7 and 8 months of age; however, testosterone levels in both vaccinated groups remained significantly lower than those in the NC group throughout the observation period (p<0.01).

Overall, these results indicate that immunocastration with the recombinant GnRH vaccine effectively suppressed serum testosterone levels in male goats to a degree comparable to that achieved by SC.

### Changes in libido

**Figure 3:**
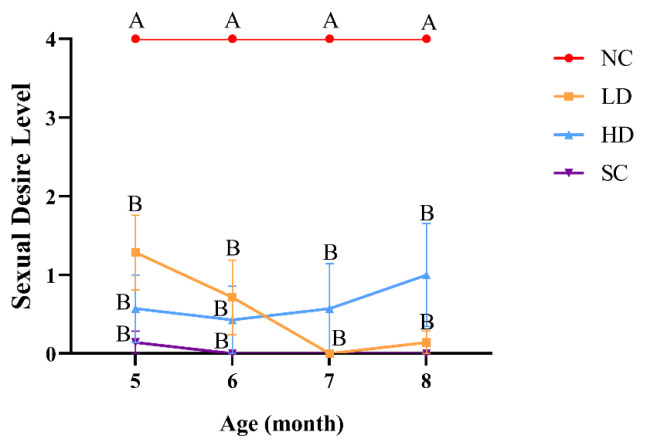
Comparison of libido scores among experimental groups. Libido was assessed using a standardized scoring system (scale: 0–4) for each experimental goat. Saline-injected control (NC), low-dose (LD), high-dose (HD), and surgical castration (SC) groups. ^A–B^ Different uppercase letters indicate p<0.01; identical letters indicate p>0.05.

As shown in Figure 3, behavioral observations indicated that goats in the NC group developed pronounced libido from 5 months of age onward, consistently maintaining a libido score of 4 throughout the observation period (5–8 months of age). In contrast, goats in the SC group and both vaccinated groups (LD and HD) exhibited markedly reduced libido scores (generally below 2) over the same period. Although the SC group tended to display slightly lower libido scores compared with the LD and HD groups, no statistically significant differences were detected among these three groups (p>0.05).

A two-way repeated measures ANOVA revealed no significant treatment×time interaction for libido scores (F = 1.90, p>0.05), suggesting that the overall temporal patterns of libido did not differ significantly among treatment groups. Despite a slight rebound in libido scores observed in the vaccinated groups after 7 months of age, values remained well below those of the NC group.

Taken together, these findings indicate that although no significant treatment×time interaction was observed, immunization with the recombinant GnRH vaccine markedly suppressed sexual behavior, producing an effect comparable to that of SC.

### Effects on testicular development

**Figure 4:**
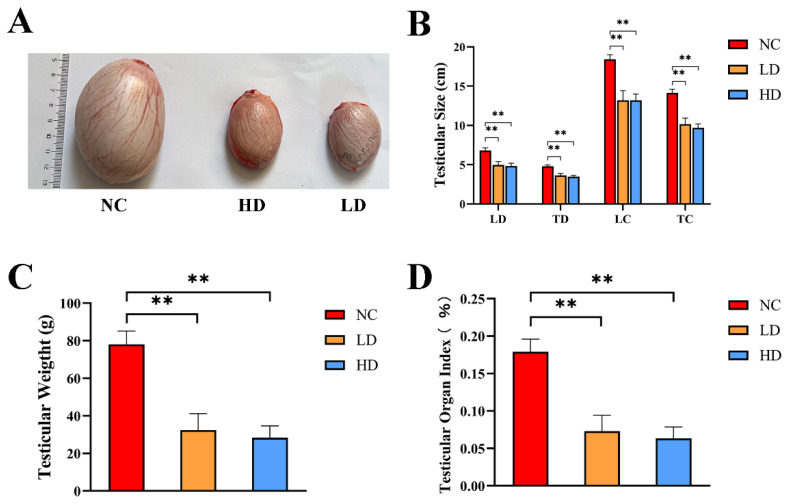
Testicular development among experimental groups at 8 months of age. (A) Representative images of testicles. (B) Testicular dimensions, including longitudinal diameter (LD), transverse diameter (TD), longitudinal circumference (LC), and transverse circumference (TC). (C) Testicular weight. Testes were weighed after removing surface blood and residual tissue, followed by phosphate-buffered saline (PBS) washing and gentle blotting to remove excess moisture. (D) Testicular organ index, calculated as testicular weight (g)/body weight (kg)×100%. ** indicates p<0.01. NC, saline-injected control; HD, high-dose; LD, low-dose.

Measurements of testicular size and weight revealed that immunization with the GnRH vaccine significantly inhibited testicular development (Figure 4). Goats in the negative control group (NC) had visibly larger testes compared to those in the vaccinated groups (LD and HD), while no obvious differences in testicular size were observed between the LD and HD groups (Figure 4A).

Detailed measurements (Figure 4B) showed that the longitudinal and transverse diameters, as well as the circumferences of the testes, were significantly greater in the NC group compared to the vaccinated groups (p<0.01). However, no significant differences were detected between the LD and HD groups (p>0.05).

Further analysis of testicular weight (Figures 4C, 4D) demonstrated that both the absolute testicular weight and the organ index (testicular weight/body weight×100%) were significantly higher in the NC group than in the vaccinated groups (p<0.01), with no significant differences observed between the LD and HD groups (p>0.05).

These findings indicate that the recombinant GnRH vaccine designed in this study effectively inhibited normal testicular development in male goats.

### Effects on testicular histological structure

**Figure 5:**
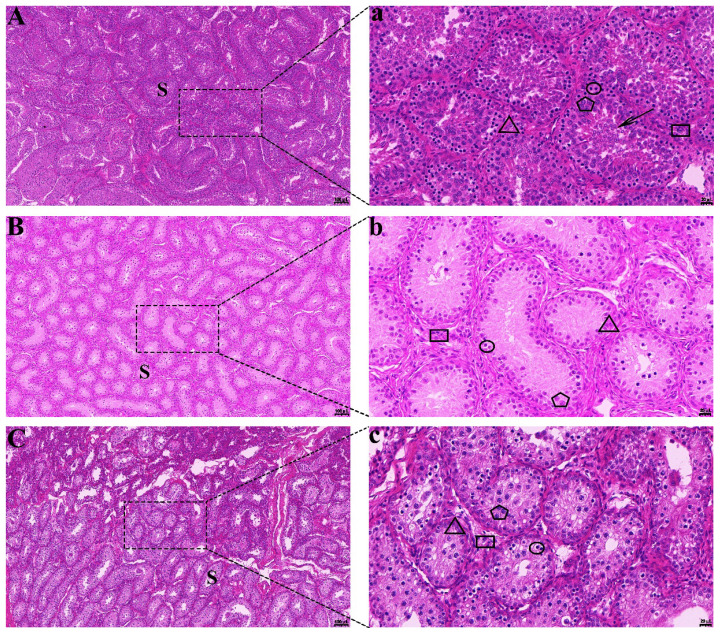
Histological examination of testicular tissue in goats at 8 months of age. Testicular tissues from saline-injected control (NC), low dose immunization (LD), and high dose immunization (HD) groups were collected and stained with hematoxylin and eosin (H&E) for microscopic observation. (A) NC group (10× magnification); (a) NC group (40× magnification); (B) LD group (10×); (b) LD group (40×); (C) HD group (10×); (c) HD group (40×). S, seminiferous tubules; rectangle, interstitial (Leydig) cells; triangle, Sertoli cells; oval, spermatogonia; pentagon, primary spermatocytes; arrow, spermatozoa.

Histological examination of testicular tissues revealed well-organized seminiferous tubules with intact architecture in the NC group, characterized by complete spermatogenic layers and abundant mature spermatozoa within the lumens (Figure 5). Interstitial cells, Sertoli cells, and spermatogenic cells at various developmental stages were morphologically normal and densely distributed.

In contrast, marked histopathological alterations were observed in the immunocastration groups (LD and HD), including pronounced atrophy of seminiferous tubules and severe disruption of spermatogenic architecture (Figure 5). No mature spermatozoa were detected in the lumens of seminiferous tubules in either immunized group, indicating effective suppression of spermatogenesis.

**Figure 6:**
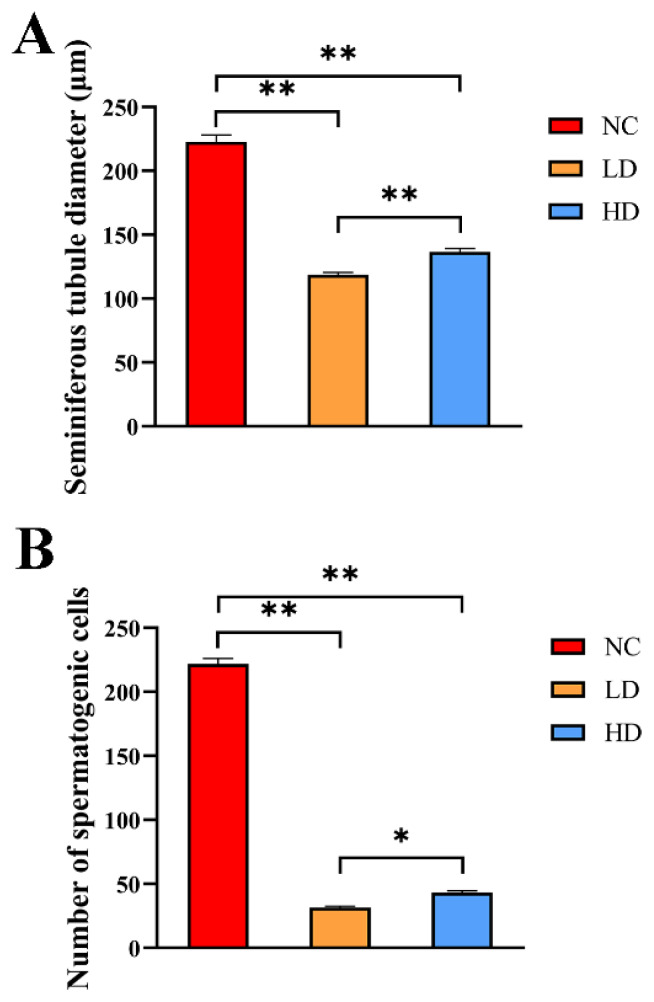
Quantitative histological analysis of seminiferous tubules following immunocastration. (A) Diameter of seminiferous tubules in the saline-injected control (NC), low-dose (LD) and high-dose (HD) groups. For each group, 30 nearly circular seminiferous tubule cross-sections were randomly selected from hematoxylin and eosin (H&E)-stained testicular sections, and the longest diameter of each tubule was measured. (B) Number of spermatogenic cells per seminiferous tubule cross-section in the NC, LD, and HD groups. For each group, 30 nearly circular seminiferous tubule cross-sections were randomly selected. Spermatogenic cells were counted based on the presence of intact nuclei within the tubules. Spermatogenic cells included primary spermatocytes, secondary spermatocytes, and both immature and mature spermatids. * indicates p<0.05; ** indicates p<0.01.

Quantitative histological analysis further confirmed these observations (Figure 6). The diameter of seminiferous tubules was significantly reduced in both LD and HD groups compared with the NC group (p<0.01 for both comparisons), and the LC group exhibited a further significant decrease compared with the HC group (p<0.01) (Figure 6A).

Similarly, the number of spermatogenic cells per seminiferous tubule cross-section was markedly decreased in the immunocastrated groups relative to the NC group (both p<0.01). Moreover, spermatogenic cell number was significantly lower in the LC group than in the HC group (p<0.05) (Figure 6B).

Together, these qualitative and quantitative histological alterations were consistent with the significant reduction in testicular size observed in immunocastrated goats, demonstrating a dose-dependent inhibitory effect of immunocastration on testicular structure and spermatogenic capacity.

### Changes in growth performance

**Figure 7:**
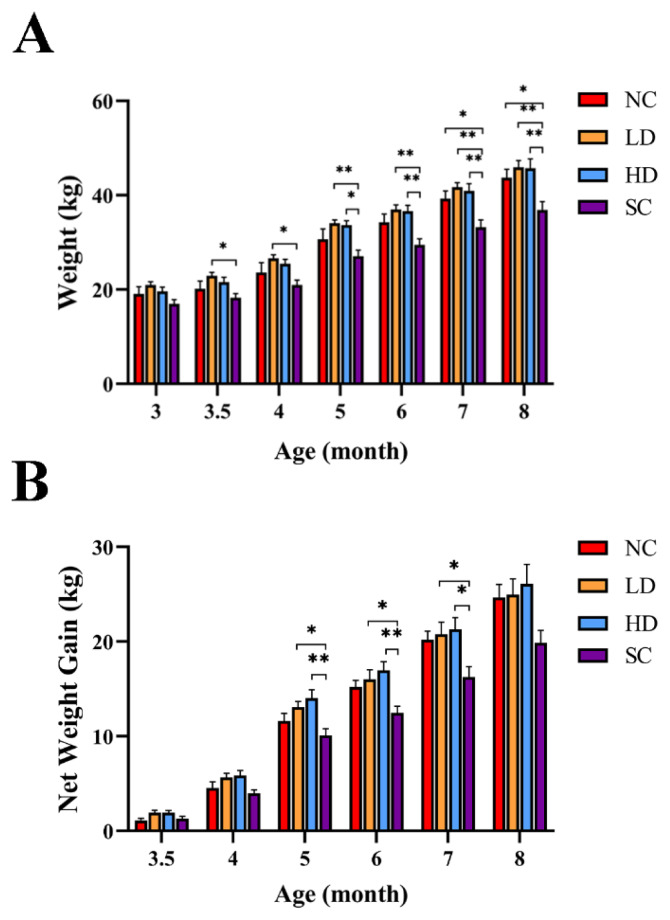
Body weight changes in goats from different treatment groups. Body weights of bucks from the saline-injected control (NC), low-dose immunization (LD), high-dose immunization (HD), and surgical castration (SC) groups were recorded at multiple time points. (A) Longitudinal body weight changes across all groups throughout the experimental period. (B) Net body weight gain relative to the baseline at 3 months of age (Calculated as weight at each time point minus weight at 3 months). * indicates p<0.05; ** indicates p<0.01.

As shown in Figure 7, analysis of growth performance indicated that GnRH immunocastration significantly influenced body weight development in male goats. A two-way repeated measures ANOVA revealed a significant treatment × time interaction for body weight (F = 2.65, p<0.05), whereas no significant interaction was observed for net weight gain (F = 2.22, p>0.05).

Body weight monitoring showed that goats in the LD group maintained significantly higher body weights than those in the SC group from 3.5 months of age until the end of the trial (p<0.05 or p<0.01). Similarly, the HD group exhibited higher body weights than the SC group from 5 months onward (p<0.05 or p<0.01). The NC group also displayed significantly higher body weights than the SC group from 7 months of age (p<0.05). Throughout the monitoring period, the SC group consistently exhibited the lowest body weight and was significantly lighter than the other three groups at most time points (p<0.05).

In contrast, net weight gain was primarily influenced by time rather than treatment, with no significant treatment × time interaction detected. Although goats in the LD and HD groups showed numerically greater net weight gain than those in the SC group at certain stages of growth, these differences were not consistent across the entire observation period. The NC group exhibited a comparable pattern, with net weight gain similar to that of the immunocastrated groups and without a significant overall treatment effect.

Overall, GnRH immunocastration did not induce the sustained growth suppression observed after SC, whereas net weight gain was not consistently affected.

## DISCUSSION

### Antibody response and optimal dose effects

Antibody responses are widely recognized as a key indicator for evaluating the effectiveness of immunocastration vaccines. Monitoring the dynamics of anti-GnRH antibody levels following immunization provides valuable information on the magnitude and duration of the humoral immune response, thereby allowing assessment of the time-dependent efficacy of vaccination. In the present study, both the LD HD groups developed detectable anti-GnRH–specific antibodies within two weeks after primary immunization. Antibody levels in both groups increased rapidly following the initial vaccination and were further augmented after the booster immunization, reaching peak values approximately two months after the primary immunization. This pattern is indicative of a robust secondary immune response, after which antibody levels gradually declined while remaining at relatively high levels, consistent with previous reports [14].

Although differences in antibody magnitude were observed between the LD and HD groups at specific time points, both groups exhibited broadly comparable antibody kinetics throughout the study period. Notably, higher antibody levels were observed in the LD group at certain sampling points, indicating that increasing antigen dose did not result in a proportionally enhanced humoral response. These findings suggest that while both doses were immunogenic, antibody magnitude did not increase linearly with antigen dose.

Similar observations have been reported in previous studies, which suggest that excessively high antigen doses may be associated with relatively reduced antibody levels compared with lower doses, despite the induction of measurable immune responses [15]. Importantly, these studies do not indicate a loss of immunogenicity at higher antigen doses. The biological basis for this phenomenon has been explored in earlier immunological studies, which propose that lower antigen doses may preferentially activate high-avidity T cells, whereas higher doses may trigger regulatory mechanisms that attenuate humoral responses [16,17]. Consistent with this concept, Uhr and Möller demonstrated that excessive antigen exposure could suppress antibody production, and Heyman reported that high antigen or IgM doses inhibited antibody responses to sheep red blood cells, whereas lower doses enhanced humoral immunity [18].

In the present study, immune tolerance or related regulatory mechanisms are proposed only as a potential explanation for the observed dose-related differences in antibody levels, rather than as evidence of impaired immunogenicity in the HD group. Given that the current data provide indirect evidence only, the involvement of such mechanisms cannot be directly confirmed. Further studies incorporating cellular immune analyses, such as T cell activation status or regulatory T cell responses, will be required to clarify the underlying mechanisms. Nevertheless, these findings underscore the importance of optimizing antigen dose to achieve robust and sustained antibody responses in GnRH-based immunocastration vaccines.

### Inhibition of testicular development and reproductive function

Puberty is a critical window for testicular development and reproductive capacity [19]. In this study, immunization was performed at 3 months of age to intervene in the hypothalamic-pituitary-testicular (HPT) axis and suppress the establishment of testicular function. Anti-GnRH antibodies block endogenous GnRH binding to its receptor, inhibiting downstream FSH and LH secretion [20]. FSH is essential for spermatogenesis and Sertoli cell function, while LH stimulates Leydig cells for testosterone production [5]. Hence, reduced secretion of both hormones disrupts testicular growth and spermatogenesis [21].

Here, LD and HD groups showed significantly reduced testicular size and weight compared to NC, with histological evidence of seminiferous tubule atrophy and absence of mature sperm. These results align with prior reports [22] and confirm that immunocastration effectively inhibits testicular development and reproductive capacity.

### Suppression of testosterone and libido

Testosterone plays a central role in regulating male sexual behavior. In the present study, serum testosterone levels in both the LD and HD groups decreased to below the detection limit from 5 months of age onward, which coincided with marked reductions in libido scores. Compared with the NC group, vaccinated goats exhibited sustained suppression of sexual activity throughout most of the observation period. However, functional endocrine suppression did not strictly coincide with the initial rise in antibody levels.

Although anti-GnRH antibody levels increased following the primary immunization, marked suppression of serum testosterone was not observed until after the booster immunization. A transient decrease in testosterone was also observed in the NC group at 6 months of age, the cause of which remains unclear; seasonal or environmental influences cannot be excluded, and this fluctuation was not accompanied by corresponding changes in libido. The delayed endocrine response in vaccinated goats is consistent with the time-dependent regulation of the HPG axis, whereby effective neutralization of pulsatile GnRH signaling likely requires sufficiently high antibody titers. The booster immunization may therefore have enhanced functional disruption of GnRH signaling, leading to sustained suppression of testosterone production.

Differences in libido dynamics were observed between the two vaccine doses. The LD group showed a gradual and persistent decline in libido, whereas the HD group exhibited more pronounced early suppression followed by partial recovery at 7–8 months of age. Although this pattern coincided with declining antibody levels in the HD group, the temporal association was not consistent across all time points, suggesting that the relationship between antibody dynamics and behavioral recovery is indirect and may involve a lag between humoral, endocrine, and behavioral responses.

Similar associations between waning anti-GnRH antibody responses and resumption of gonadal function have been reported previously [23]. Accordingly, while the present findings support a dose- and time-dependent effect of GnRH immunization, the mechanistic link between antibody decline and libido recovery remains hypothetical and warrants further investigation. These observations underscore the importance of optimizing immunization dose and timing to achieve sustained suppression of reproductive function.

### Growth performance advantages of immunocastration

Analysis of body weight data revealed that goats in the immunocastration groups (LD and HD) exhibited body weight development comparable to that of the NC group throughout most of the study period, while consistently showing higher body weights than the SC group, particularly during the later growth stages. Although the HD group displayed numerically greater body weight increases at certain time points, overall growth performance did not differ markedly among the NC, LD, and HD groups (Figure 7A). These findings indicate that immunocastration preserved normal growth performance comparable to that of intact males and, importantly, avoided the sustained growth suppression observed following SC, rather than enhancing growth beyond normal physiological levels. This pattern is unlikely to be explained solely by reduced behavioral energy expenditure, as goats in the NC group also exhibited steady body weight increases throughout the experimental period.

Based on previous studies, immunocastration has been proposed to confer certain physiological advantages over SC. First, it avoids wound-related stress responses and postoperative inflammation associated with testicular removal. Second, although testosterone levels decline markedly following immunization, hormonal suppression occurs in a gradual and potentially reversible manner, in contrast to the abrupt and permanent endocrine disruption induced by SC [19]. These differences in stress exposure and endocrine dynamics may contribute to the preservation of growth performance in immunocastrated goats.

It has been hypothesized that during periods of sustained but moderate anti-GnRH antibody levels, trace amounts of circulating testosterone may persist below conventional detection limits while retaining limited biological activity, potentially supporting anabolic signaling pathways such as IGF-1 and mTOR [5]. However, because growth performance was largely comparable among the NC, LD, and HD groups in the present study, this hypothesis is not directly supported by the current data and should be interpreted with caution. Moreover, these endocrine and metabolic pathways were not directly assessed and therefore remain speculative, requiring further experimental validation.

In addition, the metabolic consequences of immunocastration may involve complex physiological adjustments beyond endocrine modulation alone. Indeed, accumulating evidence indicates that growth performance in goats reflects integrated regulation across endocrine and nutritional axes, with recent studies showing that dietary interventions can reshape metabolic efficiency and production traits [24]. Evidence from cattle studies has suggested that immunocastrated animals may exhibit improved feed conversion efficiency and carcass yield compared with surgically castrated counterparts [19]. Such advantages are likely attributable to a combination of reduced sexual behavior, attenuated neuroendocrine stress responses, and altered energy allocation, rather than a single dominant mechanism [25]. Accordingly, the growth-related effects of immunocastration should be viewed as multifactorial, and further studies integrating endocrine, metabolic, and nutritional parameters are warranted to clarify the mechanisms underlying growth performance following immunocastration.

### Structural optimization of the vaccine and prospects for industrial application

The duration of antibody persistence is also closely associated with the vaccine’s structural design strategy [26]. In this study, a recombinant vaccine was developed by fusing a multimeric GnRH peptide with a T-helper epitope (DTT) and a pMAL carrier tag. The pMAL expression vector facilitates soluble antigen production and improves antigen stability and *in vivo* presentation efficiency, which is especially beneficial for weakly immunogenic peptides like GnRH [27].

The diphtheria toxin truncated protein (DTT) has been validated as an effective T-helper epitope enhancer. Its incorporation significantly improves the immunogenicity of small antigens such as GnRH peptides. Vaccines formed by fusing DTT with GnRH peptides not only enhance antibody production [28] but also prolong antibody duration and elicit stronger humoral and cellular immune responses [29]. Furthermore, several studies have demonstrated that combining DTT-type helper epitopes with multimeric GnRH peptides can stimulate polyclonal B-cell responses, thereby increasing both antibody titers and response uniformity [30]. As a result, the vaccine construct applied in this study exhibited strong immunogenicity and sustained immune activation.

Our results showed that the LD group achieved superior antibody responses compared to the HD group, suggesting the existence of an optimal antigen dose window between immune activation and immune tolerance. Future research should focus on refining booster schedules, selecting appropriate adjuvants, and evaluating breed-specific immune differences, in order to support large-scale application of this vaccine in goat production and promote its implementation as a non-invasive immunocastration strategy.

## CONCLUSION

This study demonstrated that the recombinant GnRH subunit vaccine constructed herein effectively suppressed the reproductive function of male goats. Notably, the LD immunization group exhibited superior immunocastration efficacy compared to the HD group, indicating a more favorable immune response at lower antigen levels. Furthermore, immunocastration not only avoided negative impacts on growth performance but also, to some extent, promoted body weight gain. These findings provide important theoretical support for the development of safe, non-invasive, and animal welfare-friendly castration technologies in livestock production systems.

## Data Availability

Upon reasonable request, the datasets of this study can be available from the corresponding author.
